# Supplementary material for: Content-rich biological network constructed by mining PubMed abstracts
Source: BMC Bioinformatics. 2004 Oct 8;5:147. doi: 10.1186/1471-2105-5-147 (PMC528731; doi:10.1186/1471-2105-5-147)
Supplement: Additional File 2 — The original results of the above study (non-essential files are deleted to keep the file size under the limit set by BMC bioinformatics). [file 1471-2105-5-147-S2.bz2 › chilibotAdditionalFile2/dip50/623ID9103205E11009/html/GRPE_DNAK.html]

 


 **GRPE** and **DNAK** 
  
Found 298 abstracts in PubMed, retrieved 50.  
 

 What does Google say? 
 PDF only 
| .edu only 

---

**Interactive relationship** (e.g. stimulation, inhibition, etc)

**Neutral relationship**- This information is important for understanding the conformational changes  **GrpE**  undergoes on binding to  **DnaK** .  Ref: 12840016 J Biol Chem< MedlineTA>J Biol Chem,
- We show that the equilibrium dissociation constants for the interaction of  **GrpE**  with wtDnaK, lidless  **DnaK**  2 517, the ATPase domain 2 388, and the substrate binding fragment 393 507 are 64 16 nM, 4.0 1.5 nM, 35 10 nM, and 67 11 μM, respectively,.  Ref: 12885236 BiochemistryBiochemistry,
- Hsc62 Hsc56  **GrpE**  is probably a new Hsp70  [ **DNAK** ]  chaperone system of E. coli.  Ref: 12054669 Biochem Biophys Res Commun, 2002
- When expressed in Escherichia coli, CGE1 can functionally replace  **GrpE**  and interacts physically with  **DnaK** .  Ref: 11752390 Plant Cell, 2001
- The Hsp 70  [ **DNAK** ]  system consisting of  **DnaK** , its cofactor DnaJ, and the nucleotide exchange factor  **GrpE** .  Ref: 12369934 Curr Protein Pept Sci, 2001
- Such results strongly suggest that the 33 residue flexible N terminal tail of  **GrpE**  binds in the substrate binding pocket of  **DnaK** .  Ref: 12885236 BiochemistryBiochemistry, 2001
- Our results reveal a complex set of molecular interactions between  **DnaK**  and its cochaperone  **GrpE** .  Ref: 12885236 BiochemistryBiochemistry, 2001
- The substrate binding properties of  **DnaK**  are controlled by its two cochaperones DnaJ Hsp40 and  **GrpE** .  Ref: 12654915 J Biol Chem, 2003
- The co chaperone  **GrpE**  is essential for the activities of the Hsp70  [ **DNAK** ]  system which assists protein folding.  Ref: 12840016 J Biol Chem< MedlineTA>J Biol Chem, 2003
- Moreover, known  **DnaK**  binding oligopeptides simultaneously affect the APIase activity of  **DnaK**  and the refolding yield of denatured firefly luciferase in the presence of  **DnaK**   **GrpE**  DnaJ ATP.  Ref: 12021775 Nat Struct Biol, 2002
- The stability of the N terminal helices is linked to the presence of the C terminal compact beta domains of  **GrpE** , providing a potential mechanism for coupling of  **DnaK**  binding activity of  **GrpE**  with temperature.  Ref: 12368105 J Mol Biol, 2002
- In eubacteria and mitochondria, Hsp70  [ **DNAK** ]  chaperone activity is controlled by the nucleotide exchange factor  **GrpE** .  Ref: 11752390 Plant Cell, 2001
- Deletion of  **DnaK**  s Lid Strengthens Binding to the Nucleotide Exchange Factor,  **GrpE**  A Kinetic and Thermodynamic Analysis.  Ref: 12885236 BiochemistryBiochemistry, 2001
- The locus has the stress genes 5  **grpE**  hsp70  [ **DNAK** ]   **dnaK**  hsp40 dnaJ 3 encoding the chaperone machine components  **GrpE** , Hsp70  [ **DNAK** ]   **DnaK** , and Hsp40 DnaJ, respectively, flanked by non heat shock inducible genes, orf16 and orf11 trkA.  Ref: 11814691 Gene, 2002
- **GrpE**  is the nucleotide exchange factor for the Escherichia coli molecular chaperone  **DnaK** , the bacterial homologue of Hsp70  [ **DNAK** ] .  Ref: 12885238 Biochemistry, 2003
- **GrpE**  is the nucleotide exchange factor for the Escherichia coli molecular chaperone  **DnaK** , the prokaryotic homologue of Hsp70  [ **DNAK** ] .  Ref: 12368105 J Mol Biol, 2002
- Under heat shock conditions, the  **DnaK**  ADP protein substrate complex is thus stabilised by a reversibly inactivated  **GrpE**  protein that refolds under permissive conditions.  Ref: 11724541 J Mol Biol, 2001
- A primer extension analysis revealed that the transcription of the  **dnaK**  operon started at the nucleotide A that is located 28 bp upstream of the  **grpE**  start codon.  Ref: 12442897 Mol Cells, 2002
- A brief survey is presented of salient findings on transcription in the Archaea, focussing on stress genes of the hsp70  [ **DNAK** ]  locus, which code for the molecular chaperones Hsp70  [ **DNAK** ] , Hsp40, and  **GrpE** .  Ref: 12236465 Crit Rev Biochem Mol Biol, 2002
- Finally, we present data disclosing that RNA binding by  **DnaK** , the Escherichia coli homolog, is influenced by the activity of its co chaperones, DnaJ and  **GrpE** .  Ref: 11720291 RNA, 2001
- These results, together with the differential kinetics of peptide binding to ADP. **DnaK**  on the one hand, and to ATP. **DnaK**  or ADP. **DnaK** . **GrpE**  on the other, suggest that ligands for either domain, i.e. ATP or ADP plus  **GrpE**  for the ATPase domain and peptides for the substrate binding domain, shift the conformational equilibrium of both domains of  **DnaK**  towards the open and closed forms, respectively, in a concerted and parallel manner.  Ref: 12383055 Biochem J, 2003
- **DnaK** , the Hsp70  [ **DNAK** ]  chaperone of Escherichia coli interacts with protein substrates in an ATP dependent manner, in conjunction with DnaJ and  **GrpE**  co chaperones, to carry out protein folding, protein remodeling, and assembly and disassembly of multisubunit protein complexes.  Ref: 12237299 J Biol Chem, 2002
- ATJ11 produced in Escherichia coli stimulated the weak ATPase activity of E. coli  **DnaK** , but was unable to stimulate refolding of firefly luciferase by  **DnaK** , and inhibited refolding by  **DnaK** , DnaJ and  **GrpE** .  Ref: 11516154 Plant Mol Biol, 2001
- Thus, the long helix pair in the  **GrpE**  dimer acts as a thermosensor that, by decreasing its ADP ATP exchange activity, induces a shift of the  **DnaK** .substrate complexes toward the high affinity R state and in this way adapts the  **DnaK**  DnaJ  **GrpE**  system to heat shock conditions.  Ref: 12639955 J Biol Chem, 2003
- In turn, it was proposed that temperature dependent down regulation of the activity of  **GrpE**  may increase the time in which  **DnaK**  binds its substrates at higher temperatures.  Ref: 12885238 Biochemistry, 2003
- Binding of peptide increased the intrinsic fluorescence of both ATP. **DnaK**  and ADP. **DnaK** . **GrpE**  and rendered their spectra similar to the spectrum of ADP. **DnaK**  with closed conformation of the ATPase domain.  Ref: 12383055 Biochem J, 2003

**Non-interactive relationship** (e.g. studied together, co-existance, homology, etc.)

- DnaJ stimulates the hydrolysis of  **DnaK**  bound ATP, and  **GrpE**  accelerates ADP ATP exchange.  Ref: 12654915 J Biol Chem, 2003
- Our simulations indicate that when the expression of recombinant protein has a low requirement for  **DnaK** , DnaJ, and  **GrpE** , the overall sigma 32 levels may drop, but the level of heat shock proteins will increase.  Ref: 11536134 Biotechnol Bioeng, 2001
- Previously, our laboratory partially characterized the  **dnaK**  operon of Streptococcus mutans hrcA  **grpE**   **dnaK**  and demonstrated that  **dnaK**  is up regulated in response to acid shock and sustained acidification G.  Ref: 11567008 J Bacteriol, 2001
- D Peptides as inhibitors of the  **DnaK**  DnaJ  **GrpE**  chaperone system.  Ref: 12637539 J Biol Chem, 2003
- SPR experiments revealed that  **GrpE**   **DnaK**  393 507 complex formation is inhibited by added peptide and abolished when the 33 residue flexible tail of  **GrpE**  is deleted.  Ref: 12885236 BiochemistryBiochemistry, 2003
- The APIase activity shows both regio and stereo selectivity and is stimulated two fold in the presence of the complete  **DnaK**   **GrpE**  DnaJ ATP refolding system.  Ref: 12021775 Nat Struct Biol, 2002
- With disulfide stabilized  **GrpE** , the rate of ADP ATP exchange and conversion of  **DnaK**  from its ADP liganded high affinity R state to the ATP liganded low affinity T state continuously increased with increasing temperature.  Ref: 12639955 J Biol Chem, 2003
- In Escherichia coli, disaggregation is carried out by a network of ATPase chaperones consisting of a  **DnaK**  core, assisted by the cochaperones DnaJ,  **GrpE** , ClpB, and GroEL GroES.  Ref: 11580258 J Struct Biol, 2001
- The effect of overproduction of the Hsp70  [ **DNAK** ]  system proteins  **DnaK** , DnaJ,  **GrpE**  and or ClpB Hsp100 from plasmids on the process of formation and removal of heat aggregated proteins from Escherichia coli cells the S fraction was investigated by sucrose density gradient centrifugation.  Ref: 11731148 FEMS Microbiol Lett, 2001
- Changes in the intrinsic fluorescence of  **DnaK**  offer a simple means not only to follow the binding of ATP and of ADP plus the co chaperone  **GrpE**  to the ATPase domain, but also to investigate the kinetics of peptide binding to the substrate binding domain of ATP. **DnaK**  and  **GrpE**  liganded ADP. **DnaK** .  Ref: 12383055 Biochem J, 2003
- Thermosensor Action of  **GrpE**  THE  **DnaK**  CHAPERONE SYSTEM AT HEAT SHOCK TEMPERATURES.  Ref: 12639955 J Biol Chem, 2003
- In the presence of excess nickel, NikR activated the transcription of ureA ureB hp72 73, nixA hp1077, copA2 hp1072, hpn hp1427 and hpn like hp1432 genes and repressed the expression of genes encoding proteins involved in ferric iron uptake and storage pfr hp0653, fur hp1027, frpB4 hp1512, exbB exbD hp1339 1340, ceuE hp1561, motility cheV hp616, flaA hp0601, flaB hp0115, stress responses hrcA  **grpE**   **dnaK**  hp111 110 109 and encoding outer membrane proteins omp11 hp0472, omp31 hp1469, omp32 hp1501.  Ref: 12890020 Mol Microbiol, 2003
- They provided clear evidence for a stress exerted by rice exudates, as shown by the induction of the universal stress protein gene uspA as well as genes of the heat shock regulon,  **grpE** , lon and  **dnaK** .  Ref: 12713466 Environ Microbiol, 2003
- Purified  **DnaK** , its cochaperones DnaJ and  **GrpE** , and ATP can facilitate reconstitution of functional 30S subunits under otherwise nonpermissive conditions.  Ref: 12150913 Mol Cell, 2002
- The effect of co overproduction of  **DnaK**  DnaJ  **GrpE**  and ClpB proteins on the removal of heat aggregated proteins from Escherichia coli DeltaclpB mutant cells new insight into the role of Hsp70  [ **DNAK** ]  in a functional cooperation with Hsp100.  Ref: 11731148 FEMS Microbiol Lett, 2001
- **GrpE** , a nucleotide exchange factor for  **DnaK** , also stimulated the ATPase activity of Hsc62 in the presence of Hsc56.  Ref: 12054669 Biochem Biophys Res Commun, 2002
- Putative heat shock proteins  **DnaK** , DnaJ,  **GrpE** , GroES and GroEL were among the proteins whose synthesis was induced in response to acid adaptation.  Ref: 12432952 J Mol Microbiol Biotechnol, 2002
- The BIA evaluation program allowed the calculation of the equilibrium constant of the interaction of M. mazeii TBP with the promoters of the stress genes  **grpE** , hsp70  [ **DNAK** ] , and hsp40 M, respectively and, for comparison, with the promoter of a non heat shock gene, orf16 M.  Ref: 12419300 Biochem Biophys Res Commun, 2002
- It is of considerable importance for structural  **GrpE**  research to answer the question of whether the  **GrpE**  dimer is only asymmetric while bound to  **DnaK**  or also as free dimer in solution.  Ref: 12840016 J Biol Chem< MedlineTA>J Biol Chem, 2002
- Primer extension results suggested the presence of promoters at the regions upstream of  **grpE**  and  **dnaK** .  Ref: 11479711 Arch Microbiol, 2001
- Two plasmids were employed pKJE7 carrying the  **dnaK**  dnaJ  **grpE**  genes under the control of the araB promoter and pClpB carrying the clpB gene under the control of its own promoter sigma 32 dependent.  Ref: 11731148 FEMS Microbiol Lett, 2001
- It was shown that refolding of thermoinactivated luciferases is completely determined by the  **DnaK**  DnaJ  **GrpE**  system.  Ref: 12387711 Biochemistry , 2002
- Active protein disaggregation by a chaperone network composed of ClpB and  **DnaK**  DnaJ  **GrpE**  is essential for the recovery of stress induced protein aggregates in vitro and in Escherichia coli cells.  Ref: 12828638 Mol Microbiol< MedlineTA>Mol Microbiol, 2002
- However, in these conditions, the Escherichia coli  **GrpE**  dimer is asymmetric since the monomer that binds  **DnaK**  adopts an open conformation.  Ref: 12840016 J Biol Chem< MedlineTA>J Biol Chem, 2002
- The HscC ATPase was not affected by the nucleotide exchange factor of  **DnaK**   **GrpE**  and stimulated 8 fold by DjlC, a DnaJ protein with a putative transmembrane domain, but not by other DnaJ proteins tested.  Ref: 12183460 J Biol Chem, 2002
- Western analysis identified five E. coli molecular chaperones including  **DnaK** , DnaJ,  **GrpE** , GroEL, and GroES.  Ref: 11858724 Protein Expr Purif, 2002
- Experiments were geared toward understanding the influence of  **DnaK**  s three domains, the ATPase residues 1 388, substrate binding residues 393 507, and lid residues 508 638 domains, on complex formation with  **GrpE** .  Ref: 12885236 BiochemistryBiochemistry, 2002
- Our results demonstrate for the first time that the ClpB  **DnaK**  DnaJ  **GrpE**  system is involved in protein monomerization and in the activation of a DNA replication factor.  Ref: 11889118 J Biol Chem, 2002
- A brief survey is presented of salient findings on transcription in the Archaea, focussing on stress genes of the hsp70  [ **DNAK** ] , Hsp40, and  **GrpE** .  Ref: 12540196 Crit Rev Biochem Mol Biol, 2002
- In this work we demonstrate that the combined action of ClpB and the  **DnaK** , DnaJ, and  **GrpE**  chaperones leads to the activation of DNA replication of the broad host range plasmid RK2.  Ref: 11889118 J Biol Chem, 2002
- Temperature directly controls functional properties of the  **DnaK**  DnaJ  **GrpE**  chaperone system.  Ref: 12639955 J Biol Chem, 2003
- Such a function has been suggested for the  **DnaK**  system of chaperones  **DnaK** , DnaJ,  **GrpE** .  Ref: 12810912 RNA< MedlineTA>RNA, 2003
- that is, sigma 32 becomes immediately available to mediate a stress response because it exists primarily in a sequestered, inactive form, complexed with chaperones  **DnaK** , DnaJ, and  **GrpE** .  Ref: 11536134 Biotechnol Bioeng, 2001
- campestris 17 revealed five ORFs whose deduced amino acid sequences show strong similarities to the bacterial HrcA,  **GrpE** ,  **DnaK** , DnaJ, and PdxK.  Ref: 11479711 Arch Microbiol, 2001
- ClpB from Escherichia coli is a member of a protein disaggregating multi chaperone system that also includes  **DnaK** , DnaJ, and  **GrpE** .  Ref: 12139937 J Mol Biol, 2002
- The genes were clustered in the transcription order  **grpE**   **dnaK**  dnaJ.  Ref: 12442897 Mol Cells, 2002
- The recombinant E. coli strain simultaneously expressing the genes for CHMO, GroEL GroES and  **DnaK**  DnaJ  **GrpE**  showed a specific CHMO activity of 111 units g 1 cell protein, corresponding to a 38 fold enhancement in CHMO activity compared with the control E. coli strain expressing the CHMO gene alone.  Ref: 12827321 Appl Microbiol Biotechnol< MedlineTA>Appl Microbiol Biotechnol, 2002
- Hsp70  [ **DNAK** ]   **DnaK**  is part of the molecular chaperone machine together with Hsp40 DnaJ and  **GrpE** , and Hsp60 is a component of the chaperonin complex.  Ref: 12747562 Adv Biochem Eng Biotechnol, 2003
- For the  **DnaK**  mediated increase in the folding yield in vitro, the minimal model for APlase catalysis involves the catalyzed partitioning of a rapidly formed folding intermediate as could be inferred from the  **DnaK**  DnaJ  **GrpE**  ATP assisted refolding of GdmCl denatured luciferase.  Ref: 12553723 Biol Chem, 2002
- Since the  **grpE**  and  **dnaK**  promoters possess E. coli sigma 32 promoter like sequences, they are functional in E. coli, although at levels much lower than in X. campestris pv.  Ref: 11479711 Arch Microbiol, 2001
- The low resolution structure of human  **GrpE**  presented here suggests that  **GrpE**  is a symmetric dimer when not bound to  **DnaK** .  Ref: 12840016 J Biol Chem< MedlineTA>J Biol Chem, 2001
- The association rate of the non heat shock gene orf16 was 0.4x10Ms and those for the stress genes,  **grpE** , hsp70  [ **DNAK** ] , and hsp40 were higher 2..5, and 3.5x10Ms, respectively.  Ref: 12419300 Biochem Biophys Res Commun, 2002
- The four heat shock genes are organized in the order hrcA  **grpE**   **dnaK**  dnaJ, a genome organization found in many gram positive bacteria, but only in one gram negative species Xylella fastidiosa.  Ref: 11479711 Arch Microbiol, 2001
- Monitoring the S fraction during co production of  **DnaK**  DnaJ  **GrpE**  and ClpB in the DeltaclpB mutant revealed that both the levels of expression and the ratios of ClpB to Hsp70  [ **DNAK** ]  system proteins had a significant effect on the formation and removal of protein aggregates in heat shocked E. coli cells.  Ref: 11731148 FEMS Microbiol Lett, 2001
- Small angle X ray scattering analysis shows the protein possesses an elongated shape in solution, and demonstrates that its envelope, determined by an ab initio method, is similar to the high resolution envelope of Escherichia coli  **GrpE**  bound to  **DnaK**  obtained from single crystal X ray diffraction.  Ref: 12840016 J Biol Chem< MedlineTA>J Biol Chem, 2001
- This unique mode of binding between  **GrpE**  s tail and  **DnaK**  contributes to, but does not fully explain, the decrease in K d from 64 to 4 nM upon deletion of  **DnaK**  s lid.  Ref: 12885236 BiochemistryBiochemistry, 2001
- This induction requires both the co chaperone activity of DjlA, in cooperation with  **DnaK**  and  **GrpE** , and its unique transmembrane TM domain.  Ref: 12655402 Mol Genet Genomics, 2003
- The possibility that deletion of  **DnaK**  s lid creates a more symmetrically shaped molecule, with enhanced affinity to  **GrpE** , is also discussed.  Ref: 12885236 BiochemistryBiochemistry, 2003
- **DnaK** , a Hsp70  [ **DNAK** ]  homolog of Escherichia coli, together with its co chaperones DnaJ and  **GrpE**  protects denatured proteins from aggregation and promotes their refolding by an ATP consuming mechanism.  Ref: 12637539 J Biol Chem, 2003
- Northern blot analyses with  **grpE** ,  **dnaK** , and dnaJ specific probes revealed that the three genes are co transcribed as a 4.0 kb mRNA.  Ref: 12442897 Mol Cells, 2002
- Addition of ATP or of ADP plus  **GrpE**  to nucleotide free  **DnaK**  resulted in a similar decrease in intrinsic fluorescence, indicating similar open conformations of the ATPase domain under these two conditions.  Ref: 12383055 Biochem J, 2003
- The overall identities in the deduced amino acid sequences of  **GrpE** ,  **DnaK** , and DnaJ with those of the Escherichia coli homologs were 45. 4.5, and 61.2%, respectively.  Ref: 12442897 Mol Cells, 2002
- Using purified protein components we show that the ClpB  **DnaK**  DnaJ  **GrpE**  synergistic action activates the plasmid RK2 replication initiation protein TrfA by converting inactive dimers to an active monomer form.  Ref: 11889118 J Biol Chem, 2002
- Here we report that d peptides efficiently inhibit the refolding of denatured luciferase by the  **DnaK**  DnaJ  **GrpE**  chaperone system EC50 = 1 2 microM.  Ref: 12637539 J Biol Chem, 2003
- campestris 17  **grpE**  and  **dnaK**  promoters were induced two to three fold over controls.  Ref: 11479711 Arch Microbiol, 2001
- In this study, we have used surface plasmon resonance SPR and isothermal microtitration calorimetry ITC to study the mechanism of complex formation between the Hsp70  [ **DNAK** ]  molecular chaperone,  **DnaK** , and its cochaperone,  **GrpE** , which is a nucleotide exchange factor.  Ref: 12885236 BiochemistryBiochemistry, 2001
- Characterization of stress responsive genes, hrcA  **grpE**   **dnaK**  dnaJ, from phytopathogenic Xanthomonas campestris.  Ref: 11479711 Arch Microbiol, 2001
- The cloned  **grpE** ,  **dnaK** , and dnaJ genes had open reading frames of 47, 926, and 16 nucleotides, coding for proteins with calculated molecular masses of 1 9 61, and 4 50, respectively.  Ref: 12442897 Mol Cells, 2002
- In the presence of excess ClpB, an increase in the levels of  **DnaK** , DnaJ and  **GrpE**  was required to prevent aggregate formation upon heat shock or to efficiently remove protein aggregates after heat shock.  Ref: 11731148 FEMS Microbiol Lett, 2001
